# Functional characterization of a bovine luteal cell culture model: Effects of passage number

**DOI:** 10.1371/journal.pone.0334047

**Published:** 2025-11-19

**Authors:** Arpna Sharma, Jens Vanselow, Christa Kühn, Doreen Becker

**Affiliations:** 1 Research Institute for Farm Animal Biology (FBN), Dummerstorf, Germany; 2 Department of Animal Sciences, University of Illinois Urbana-Champaign, United States of America; 3 Friedrich Loeffler Institute, Federal Research Institute for Animal Health, Greifswald, Insel Riems, Germany; Federal University of Minas Gerais: Universidade Federal de Minas Gerais, BRAZIL

## Abstract

Primary cell culture models are useful tools to analyze intracellular signaling pathways and interactions in detail. However, at higher passages, vital cell characteristics such as cell morphology, physiology, gene expression and cell proliferation can be compromised which may result in variable data. In the present study, we characterized cultured primary luteal cells (PLCs) and compared them to intermediate passaged luteal cells i.e. passage number 15 (P15) and high passaged luteal cells i.e. passage number 30 (P30). To explore *in-vitro* culture induced variabilities, PLCs were passaged repeatedly until passage number P30. Expression of cell cytoskeleton proteins was monitored by immunofluorescence and steroidogenic acute regulatory (STAR) protein expression was detected by capillary electrophoresis as physiological key parameter. The abundance of STAR, hydroxy-delta-5-steroid dehydrogenase, 3 beta- and steroid delta-isomerase 1 (HSD3B1) and luteinizing hormone/choriogonadotropin receptor (LHCGR) marker transcripts was quantified by RT-qPCR. Cell proliferation and cell viability of luteal cells were evaluated using flow cytometry. Global gene expression profiling by RNA sequencing was performed on early passaged (P3) luteal cells. Cell passaging severely reduced the expression of genes encoding marker proteins of luteal cells. Similarly, progesterone (P4) synthesis and cell proliferation were reduced significantly at higher passages. Early passaged (P3) luteal cells expressed key genes of luteal cells but with lower expression values. Luteal cells remained highly viable and consistently co-expressed both vimentin and cytokeratin-18 protein in their cytoskeleton irrespective of passage number. Together, these findings demonstrate that only short-term luteal cell primary cultures or very early passaged luteal cells (P3) are able to display molecular features that resemble that of the corpus luteum *in vivo* and thus are suitable *in vitro* models.

## Introduction

*In vitro* cell culture models are indispensable for studying key physiological functions of organ-specific tissues. However, *in vitro* cultured cells pose a limitation in their inability to comprehensively capture the inherent organ-specific characteristics. This leads to changes in their phenotype and genotypic properties especially during repeated passaging [1,2]. Passage number is an important factor which informs about the *in vitro* age of cells and is essential for evaluating cell growth and their functional status [3]. Until a definite passage number and days, hormonally active long-term cultures of human ovarian stromal cells and luteal cells have been characterized to elucidate their physiological function *in vitro* [4,5]. Similarly, long-term cultures of bovine granulosa cells have been established to study the function of differentiation and proliferation [6]. Highly proliferative cells of organs such as kidney [7], liver [8] and pancreas [9] have been long-term cultured but lose their cell-type specific functions *in vitro.* Similarly, immortalized cell lines have gathered wide usage as unlimited *in vitro* models for experimental purposes in cancer research and drug testing [10]. However, serial passaging of cell lines also causes genotypic and phenotypic variations [11] which could lead to unreliable experimental outcomes. And several studies have reported significant variability in the behaviour of different cell types due to repeated passaging [12–14]. In the bovine functional ovary, corpus luteum has a high cell renewal potential *in vivo* [15] and luteal cells have immense applicability in dairy cattle clinical studies [16]. Bovine luteal cells have been characterized *in vitr*o although as short-term cultures [17,18]. Bovine luteal cells co-cultured with early embryos have been utilized to study their steroidogenic and prostanoid crosstalk [19]. Also, across several other species, PLCs have been extensively used in co-culture *in vitro* models to functionally improve the experimental outcomes of cells under investigation [20–22]. However, to our knowledge there are no reports available on effects of repeated passaging on the functionality of luteal cells. This research question is relevant, as it would reveal if repeatedly passaged luteal cells are suitable for cell based *in vitro* experiments. Therefore, in the present study *in vitro* physiological characteristics of PLCs were compared to intermediate passaged P15 and high passaged P30 luteal cells.

## Materials and methods

### Ethics statement

No animal experiments were performed in this study. The corpus luteum was excised after slaughter of a Holstein cow on 11.06.2020 at the experimental abattoir of the Research Institute for Farm Animal Biology (FBN) Dummerstorf, Germany. The 39-month-old, Holstein-Friesian cow was born on 20.03.2017 and had delivered a calf on 26.01.2020 and was in her 2^nd^ lactation of her 137^th^ day in lactation.

### Isolation and culture of luteal cells

The corpus luteum was fully developed [23] and was excised from the ovary and washed in 1x phosphate buffered saline (PBS) and cut into small pieces using a sterile scalpel. Next, small luteal tissue pieces were digested in 20 ml digestive solution (Hanks’ Balanced Salt Solution (HBSS-Hepes+antibiotic mix)) including 200 µl (0.1%) of collagenase (NB4G 17465 SERVA 10%) for 25 minutes (min.) at 37°C. This step was repeated at least 4 times and each time the filtrate (20 ml) was passed through a sterile cell sieve (100µm) to remove undigested tissue chunks. The filtrate was then transferred into a 50 ml tube containing 20 ml of DMEM/HAM’s F12 (Bio&Sell, BS.F4815) + 5% fetal bovine serum (FBS) (Bio&Sell, BS.S0615) and centrifuged at 400g for 5 min (Allegra x-12R centrifuge, Beckman-Coulter, Krefeld, Germany) to sediment the cells, which were then re-suspended in 5 ml DMEM/HAM’s F12 + 5% FBS. The supernatant was again supplemented with additional medium and centrifuged at 400g for 5 min. to recover additional cells. Finally, all cell suspensions were pooled and centrifuged at 400g for 5 min. The cell pellet was then dissolved in 20 ml DMEM/HAM’s F12 + 5% FBS + 2 mg DNAse 1, and again centrifuged at 400g for 5 min. Then, the pellet was dissolved in 3–6 ml of DMEM/HAM’s F12 + 5% FBS. Next 3 ml of cell suspension was loaded on a discontinuous Percoll (Biochrom L6143) density gradient with densities of 1.02, 1.03, 1.04 and 1.05 and allowed to centrifuge for 20 min at 1697g without brake. The gradients were then peeled off one by one and interphases 1.03 and 1.04 were selected and resuspended in DMEM/HAM’s F12 + 5% FBS medium and centrifuged at 400g for 5 min. Next, cell pellet was re-suspended in the freezing medium (10% DMSO + 90% FBS) and cells were cryopreserved and stored in liquid nitrogen (−196°C) for later culture and analysis. A fresh cryovial of approximately 2.2x10^6^ PLCs were cultured in collagen (0.2% collagen R, SERVA, Cat.No. 47254) coated 6-well culture plates (TPP^®^ tissue culture plates) in DMEM/HAM’s F12 (Bio&Sell, BSF4815) + 5% FBS culture media supplemented with luteinizing hormone (2U/ml, Ovogest®300 I.E./ml from MSD Animal Health), Insulin-Transferrin-Selenium (ITS) (Sigma, 1x I3145), epidermal growth factor (EGF) (10ng/ml, Sigma E9644), L-Glutamine (2.5mM, Bio&Sell BS.K0283), penicillin/streptomycin (100 ug/ml, Bio&Sell BS.A2213) and amphotericin (0.25 ug/ml, Bio&Sell BS.A2613). Cultured PLCs at 80% confluency were washed with 1x PBS and trypsinized using 800 µl of accutase, (Sigma-Aldrich, A6964) and were sub-cultured in new 6 well culture plates. At each subculturing/passaging, luteal cells were cryopreserved, and viability was measured using trypan blue dye and cell number was counted in a Thoma cell counting chamber. For independent *in vitro* experiments, fresh cryovials of PLCs were used while for P15 and P30 luteal cells either cryovials of earlier passage were thawed in a water bath at 37°C, quickly diluted with 5 ml of culture medium (DMEM/HAM’s F12 + 10% FBS) and centrifuged at 500g for 3 min. The supernatant was then removed, and the remaining luteal cell pellet was resuspended in culture media. All independent *in vitro* experiments of PLCs, P15 and P30 luteal cells were cultured in DMEM/HAM’s F12 + 10% FBS in 24-well collagen coated culture plates and incubated at 37°C, 5% CO_2_ for 48 hours (hrs).

### Cell proliferation analysis

The proportion (%) of proliferating cells was determined by DNA fluorescence detected by flow cytometry. Briefly, around 1.1-1.3x10^5^ PLCs, P15 and P30 luteal cells were cultured in triplicates in a 24 well culture plate and cultured for 48 hrs. After 48 hrs, cells were washed with 1x PBS and subjected to trypsinization by adding 800 µl accutase (Sigma-Aldrich, A6964) to each well of the 6-well culture plate. The detached cells were centrifuged (3 min., 500g) and the remaining cell pellet was dissolved in 300µl of 1x PBS. Next, the cell suspension was slowly added dropwise into 70% ice-cold ethanol and stored at −20°C. Later, cells were pelleted (300g, 10 min., 4°C), re-suspended in 1 ml RNase solution (1 mg/ml), and incubated at 37 °C for 30 min. Then, propidium iodide (PI) reagent (500 µg/ml) was added to the cells and incubated in the dark at 37°C for 30 min. Lastly, the fluorescence signal was quantified from single cells (10,000 counts) by a flow cytometer (Gallios, Beckman-Coulter, Krefeld, Germany) and proportions of cells at different cell cycle phases were analyzed using the MultiCycle Tool of FCS Express software.

### Lipid droplet analysis

Approximately 20,000 of PLCs, P15 and P30 luteal cells were cultured independently in µ-Slide 8 Well high chamber slides (ibidi, cat.no:80806) and after 48 hrs cells were fixed in 4% paraformaldehyde (PFA) for 21 min. at 4°C. Next, cells were washed once with 1xPBS for 5 min. at room temperature (~22°C) and then 200 µl of Dye mix (1:400 Lipid Droplet Dye (SCT144, Merck), 1:1000 Phalloidin-iFluor 555S (ab176756, Abcam) and 1:2000 Hoechst33342 (10 mg/ml PBS stock solution; B2261, Sigma) were added in each well and cells were incubated for 60 min. at room temperature (~22°C). Later, cells were washed twice with 1x PBS for 5 min. at room temperature (~22°C). Lastly 400 µl of 1x PBS was added in each well and cells were subjected to fluorescent microscopy for capturing the cytoplasmic lipid droplet signal.

### Radioimmunoassay (RIA)

The P4 concentrations in spent culture media of PLCs, P15 and P30 luteal cells were determined by competitive 3H–radioimmunoassay with rabbit-raised antibodies. The P4 tracer (1,2,6,7-3H) was purchased from Hartmann Analytic. Assay standards were prepared in RIA buffer (Gelatine (0,1%)- PBS) after dissolving the tracers in 99.8% ethanol. The radioactivity levels were measured in a liquid scintillation counter with an integrated RIA-calculation programme (TriCarb 2900 TR; PerkinElmer, Germany).

### Capillary electrophoresis

Approximately 1.1−1.3x10^5^ of PLCs, P15 and P30 luteal cells were cultured in 24 well-culture plates and after 48hrs, cells were washed with 1 x PBS and lysed with 1 x MPER buffer and protein concentrations were measured using the Micro BCA Protein assay kit according to the instructions (Thermofisher Pierce Biotechnology 23235). For protein detection, the capillary western method was performed using the WES instrument system (ProteinSimple, Bio-Techne) according to the manufacturer’s instructions. Protein samples, wash buffers, blocking reagents, primary antibody (STAR, PA3−21687, Invitrogen; Beta actin, SC47778, Santa Cruz) and chemiluminescent substrates were prepared and loaded on the respective wells of the assay plate. The assay plate was loaded onto the instrument for protein separation in a 12–230 kD capillary separation module (SM-W001). The detection of protein bands was automatically performed by the instrument. The anti-mouse (DM-002) secondary antibody detection module was purchased from ProteinSimple (Bio-Techne).

### Reverse transcription and quantitative real-time PCR

Around 1.1−1.3x10^5^ of PLCs, P15 and P30 luteal cells were cultured for 48 hrs and then washed with 1x PBS and further subjected to RNA isolation using the InnuPREP RNA Mini Kit (Analytik Jena, Germany). The corresponding RNA concentrations were quantified using a NanoDrop1000 Spectrophotometer (Thermo Scientific, Bonn, Germany). For reverse transcription, 200 ng of RNA from each cell sample was utilized for cDNA preparation using SensiFAST cDNASynthesis Kit (Bioline, Luckenwalde, Germany) at 25 °C for 10 min, followed by 42 °C for 15 min and lastly at 85 °C for 5 min. Later, for gene expression analysis, external standards for each target gene were cloned in pGEM T-vector and validated by sequencing for specificity of primer pairs. Later, quantitative real-time PCR was performed using SensiFAST SYBR No-ROX (Bioline) with different dilutions of external standards along with gene-specific primers (S1 File) and in a light cycler 96 instrument (Roche, Mannheim, Germany). The relative gene expression was then determined using ribosomal protein lateral stalk subunit P0 *(RPLP0)* gene as the reference gene. *RPLP0* was selected as reference gene as it has been previously validated as ideal reference gene due to its stable expression in bovine [24] and human [25] ovarian granulosa cells as well in many human cancer cell lines [26–28].

### polyA + RNA sequencing

A cryovial of passage 2 containing approximately 1.45x10^6^ luteal cells were thawed and cultured in a 6 well culture plate containing culture media (DMEM/HAM’s F12 + 5% FBS). At reaching 80% of confluency, the plated P3 luteal cells were trypsinized and around 4 million luteal cells were harvested which were subjected to RNA isolation using the Qiagen DNA/RNA AllPrep Kit (catalogue no. 80204) following the manufacturer’s instructions. RNA was evaluated for quantity (Qbit, Thermofisher), quality (Bioanalyzer, Agilent) and traces of DNA residuals [29]. In case of DNA contamination, an extra cleaning step *via* DNAse digestion was added. RNAseq library was prepared using a strand-specific protocol (TruSeq® Stranded mRNA Library Prep Kit (Illumina)) including polyA-selection. Quality of the RNAseq library was monitored on the Bioanalyzer 2100 (Agilent). The library was paired-end sequenced for 2x 100 bp up to 141 million reads on a HiSeq2500 (Illumina). Fastq files were mapped against the ARS-UCD1.2 genome assembly complemented by the bovine Y chromosome sequence (1000 Bulls reference genome file) using the annotation file from Ensembl (v102). The analysis was performed using nf-core–Nextflow. The pipeline utilized was nf-core/rnaseq-3.4 (https://nf-co.re/rnaseq/3.4/usage). Briefly, the pipeline performs extensive quality checks, and mapping against the reference genome using STAR software. STAR was chosen over the HISAT2 aligner, because STAR works better with soft-clipped reads and mismatched bases, resulting in a higher mapping rate [30].

### Cell immunofluorescence

Cell cytoskeleton proteins such as intermediate filament protein *viz* (IFs) vimentin and cytokeratin-18 (KRT-18) were evaluated by immunofluorescence. Approximately 20,000 PLCs, P15 and P30 luteal cells, were cultured independently in µ-Slide 8 Well high chamber slides (ibidi, cat.no:80806) and after 48 hrs, cells were fixed in 4% paraformaldehyde (PFA) for 21 min. at 4°C, next washed in 1x PBS, permeabilized with 0.1% Triton X-100 for 10 min. at room temperature (~22°C) and blocked with 5% bovine serum albumin (BSA) for 30 min. at room temperature (~22°C). Further, cells were incubated with primary antibodies against vimentin (Invitrogen, catalog no. MA5–11883, 1:100) and KRT-18 (Novus biologicals, catalog no. NBP2–44951, 1:100) at 4°C overnight. Next day, cells were rinsed four times in wash buffer and were incubated with goat anti-Mouse IgG (H + L) cross-adsorbed secondary antibody, alexa Fluor 647 (Invitrogen, catalog no. A21235, 1:200) in dark at room temperature (~22°C) for 1 hr. Further, cells were washed 4x for 5 min. with wash buffer to omit excess bound antibodies. Next, cells were incubated with SYBR green (1:500) in the dark at room temperature (~22°C) for 20 min. followed by 4x rinsing in wash buffer and further incubation in 2% PFA at room temperature (~22°C) for 20 min. Lastly, cell fluorescence signal was visualized with a 20x oil objective lens, and images were captured by a confocal laser scanning microscope LSM 800 assembled with ZEN software (Carl Zeiss Inc, Germany).

### Cell viability assessment

Cultured PLCs, P15 and P30 luteal cells were thoroughly washed twice with 1x PBS and then trypsinized using 200µl accutase (Sigma-Aldrich, A6964) at 37^°^C for 10 min. Post trypsinization, all detached cells were centrifuged and resuspended in 1 ml DMEM/F12 medium and analyzed for viability and apoptosis using an Annexin-V FITC/PI kit (Miltenyi biotec, Germany). Cells were centrifuged and pellets were re-suspended in 100µl of binding buffer to which 10µl of Annexin V reagent was added and kept for incubation in the dark for 15 min. followed by washing and resuspension in 500µl binding buffer. Next, 5µl of PI (Propidium iodide, 500 µg/ml) was added to the cells with gently mixing just prior to flow cytometric analysis. The fluorescence signal was quantified from single cells (10,000 counts) by the flow cytometer (Gallios, Beckman-Coulter, Germany) and the data obtained was analyzed using the Kaluza-software (Beckman-Coulter, Germany).

### Statistical analysis

The mRNA abundance, P4 estimation, cell proliferation and cell viability values from at least three independent cell culture experiments were subjected to one way ANOVA (Holm-Sidak method) in SigmaPlot 11.0 to test for any statistical significance. In case of failed equal variance test, one way ANOVA on ranks (Dunn’s Method) was performed. P value *<* 0.05 was considered statistically significant. The bar plots were created in ggplot2 R package (version 4.2.2).

## Results

### Cell doubling rates

**Fig 1:**
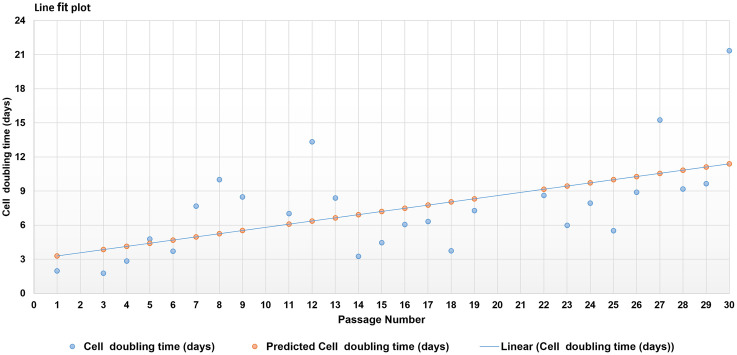
A linear regression model was applied to determine the relation between luteal cell passage numbers and doubling time. Linear regression indicated a moderate yet a direct relationship between luteal cell passage number and doubling rate. A correlation (R) value of 0.5782 with *p* value of 0.001974 signified a positive correlation between passage number and cell doubling time.

Cell population doubling time was evaluated for luteal cells from different passages (http://www.doubling-time.com/compute.php) (S1 File). Further, to assess the relationship between passage number and population doubling time, a simple linear regression model was performed (www.statskingdom.com). The significance of the regression model was evident by a *p* value of 0.001974 with a correlation (R) value of 0.5782 which indicated a moderate yet a positive correlation between passage number and luteal cell doubling time (Fig 1).

### Luteal cells proliferation decreases with successive passaging *in vitro*

**Fig 2:**
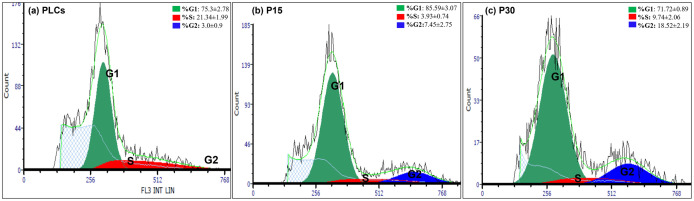
Representative cell cycle distribution histograms observed in PLCs, P15 and P30 luteal cells by flow cytometric analysis. Numbers indicate average percentage (%) in G1, S and G2/M-phase of cell cycle from at least three independent cell culture experiments from PLCs, P15 and P30, respectively. G1-phase is denoted by green peaks, S-phase by red peaks, and G2-phase by blue peaks.

Flow cytometry analyzed the cell proliferation by estimating the distribution of PLCs, P15 and P30 luteal cells in different phases of cell cycle (G1, S and G2 phase) (Table 1). As indicated in representative histograms (Fig 2a, 2b and 2c) and in Table 1, the percentage (%) of P15 luteal cells in G1-phase was significantly higher (*p < 0.05*) as compared to % of P30 luteal cells in G1-phase. The percentage of PLCs in S-phase was significantly higher (*p < 0.05*) as compared to both P15 and P30 luteal cells. This was indicative of the reduced DNA replication in cultured luteal cells with successive passaging *in vitro.* Further, the proportion of P30 luteal cells in G2 phase was significantly higher (*p < 0.05*) as compared to PLCs and P15 which suggested that luteal cells at higher passage number grow very slowly as indicated by more cell’s accumulation in G2-phase. This may lead to either delayed cell proliferation or complete cell arrest.

**Table 1:** Cell proliferation analysis of cultured PLCs, P15 and P30 luteal cells.

	G1-phase (%)	S-phase (%)	G2-phase (%)
PLCs	75.30 ± 2.78^ab^	21.34 ± 1.99^a^	3.0 ± 0.90^a^
Passage 15	85.59 ± 3.07^a^	3.93 ± 0.74^b^	7.45 ± 2.75^a^
Passage 30	71.72 ± 0.89^b^	9.74 ± 2.06^b^	18.52 ± 2.19^b^

Significant changes were recognized with one way ANOVA, if *p < 0.05*. Data represented as means±SEM, n=3.

### Luteal cell morphology and lipid droplet content are altered with subsequent passaging

**Fig 3:**
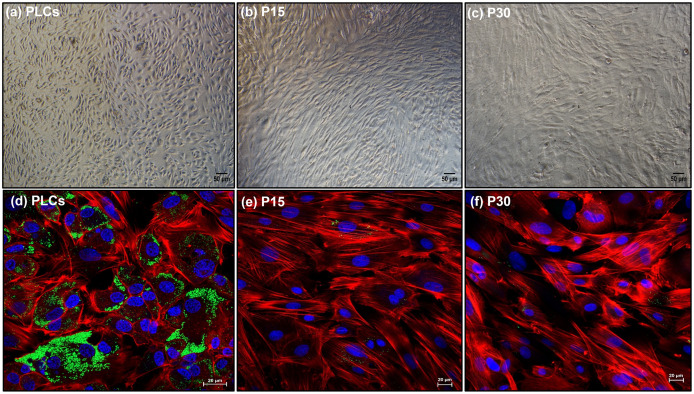
*In vitro* luteal cell morphology and lipid content: *In vitro* cultured (a) PLCs displaying normal spindle shaped morphology, and both, (b) P15 and (c) P30 luteal cells showed elongated and more flattened morphology *in vitro*, scale bar 50µm. Fluorescent imaging showing (d) PLCs loaded with large amount of lipid droplets as indicated by strong green fluorescence, while both (e) P15 and (f) P30 luteal cells showed negligible staining. Lipid droplets were stained with biotracker 488 green lipid droplet dye, actin filament stained by phalloidin-ifluor 555 (red signal) and nuclei stained by Hoechst33342 (blue signal), scale bar 20µm.

PLCs, P15 and P30 luteal cells were cultured for 48 hrs and images were captured under a phase contrast microscope. The cultured PLCs (Fig 3a) displayed an elongated morphology compared to enlarged and more flattened morphology displayed by both P15 (Fig 3b) and P30 luteal cells (Fig 3c). In addition, the qualitative measurement of lipid droplet staining using BioTracker 488 Green lipid droplet dye revealed that cultured PLCs were loaded with lipid droplets as indicated by strong green fluorescent signals in the cell cytoplasm (Fig 3d). However, with passaging the lipid droplet content in both P15 (Fig 3e) and P30 (Fig 3f) declined drastically as indicated by negligible fluorescent signal in their cell cytoplasm. Together, these results indicate that subsequent *in vitro* passaging significantly affected both luteal cell morphology and lipid content in both P15 and P30 luteal cells.

### Luteal cells lose the expression of marker genes at higher passage number

**Fig 4:**
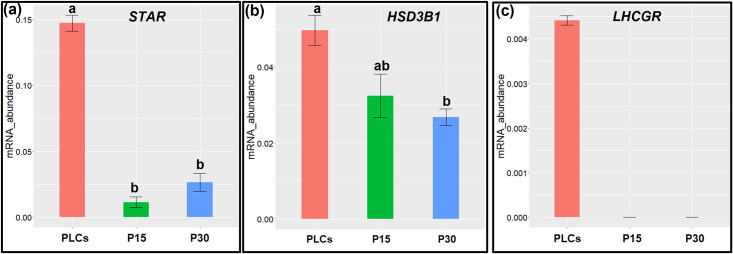
Bar plots represent expression of marker genes (a) *STAR,* (b) *HSD3B1* and (c) *LHCGR* in PLC, P15 and P30 luteal cells. Statistically significant changes were recognized with one way ANOVA, if *p < 0.05*. Data represented as means±SEM, n=3.

**Fig 5:**
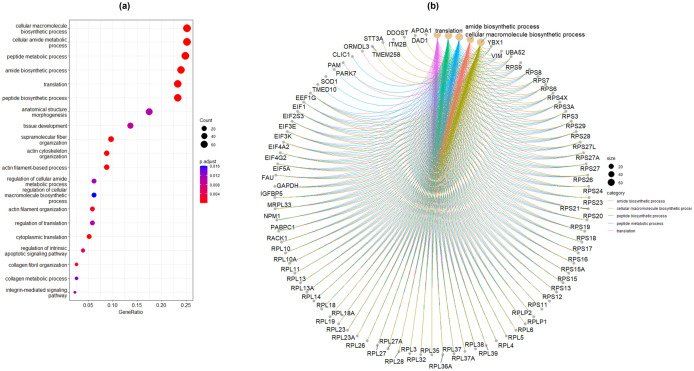
GO term illustration (a) The dotplot displaying the top 20 biological processes (adjusted *p values* <0.05) in early passaged (P3) luteal cells (b) The cnetplot depicting the gene clusters associated to top 5 biological processes. Biological process associated to each gene is displayed in a specific color code. Common genes that are closely associated between the different biological process are plotted foremost and so on. Both dotplot and cnetplot were created using clusterProfile in R.package (4.2.2).

We selected three marker genes of luteal cells, *STAR*, *LHCGR* and *HSD3B1* to analyze their mRNA expression in cultured PLCs, P15 and P30 luteal respectively. The mRNA expression of *STAR* was significantly (*p < 0.05*) higher in PLCs compared to P15 and P30 luteal cells (Fig 4a). Similarly, the mRNA expression of *HSD3B1* was significantly (*p < 0.05*) higher in PLCs compared with P30 luteal cells (Fig 4b). *LHCGR* mRNA expression was higher (*p < 0.05*) in PLCs while completely absent in P15 and P30 luteal cells (Fig 4c) (S1 File in S3). Further, RNA sequencing results of early passaged luteal cells (P3) revealed 13,763 expressed genes in luteal cells (S2 File in S4). The relative mRNA abundance of these expressed genes was represented as transcripts per million (TPM). Among them, luteal cell steroidogenesis specific genes showed an expression with TPM values of *STAR=23.46, LHCGR= 0.101* and *HSD3B1=79.74* respectively. However, contrary to these luteal marker genes, cytochrome c oxidase I (*COX1=20282.13),* cytochrome c oxidase III (*COX3=11694.77),* mitochondrial encoded ATP synthase membrane subunit 8 (*ATP8=15939.97),* collagen type I alpha 2 chain (*COL1A2=13855.16)* and secreted protein acidic and cysteine rich (*SPARC=12555.66)* were among topmost expressed genes with very high TPM expression values. Further, gene ontology (GO) enrichment analysis was performed on top 500 highly expressed genes in R(version 4.2.2) using the visualization and annotation packages *viz* clusterProfiler v4.4.4 [31], AnnotationDbi and org.Bt.e.g. db. The GO term; biological processes was allocated to gene clusters by adjusted p-value priority to 30 different biological processes (S2 File in S5). Top 20 significant biological processes have been illustrated as dotplot (Fig 5A) and genes linked to top 5 biological processes were illustrated in cnetplot (Fig 5B).

### P4 levels and STAR protein expression are affected with passaging

**Fig 6:**
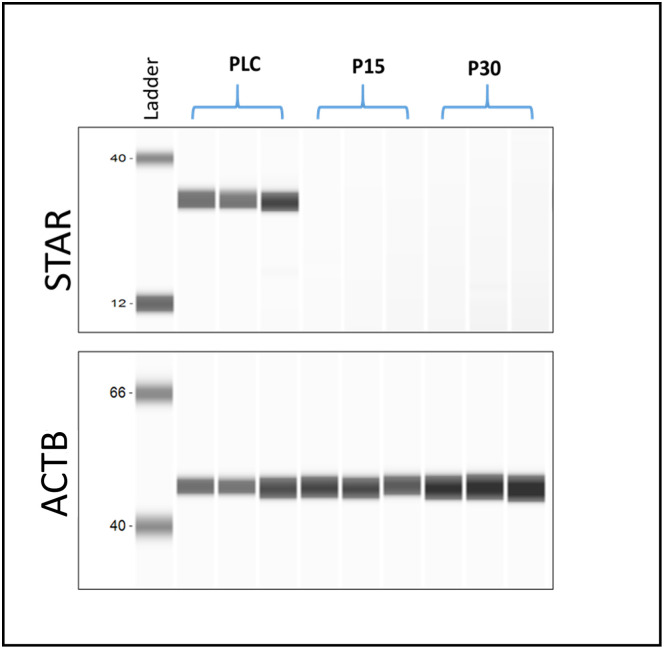
STAR protein detection by capillary electrophoresis: STAR protein detected in PLCs showed a strong band of approx. 37kDa, while in P15 and P30 luteal cells no band was detected. The housekeeping protein β-actin (ACTB) was detected at approx. 44kDa. Data image represents blot results of three independent experiments each of PLCs, P15 and P30 luteal cells.

The spent media of cultured PLCs, P15 and P30 luteal cells were subjected to RIA for P4 estimation. Results revealed relatively high amounts of P4 in spent media of PLCs (1199.57 ± 51.97 ng/ml) whereas very low amounts of P4 were detected in spent media of P15 (3.77 ± 1.66 ng/ml) and P30 (4.19 ± 1.84 ng/ml) luteal cells. To confirm the effects of passaging on the luteal cell P4 synthesis, we quantified the STAR protein expression using the Wes instrument. STAR protein initiates steroidogenesis in luteal cells by transferring cholesterol from the outer to the inner mitochondrial membrane, while HSD3B1 enzyme converts pregnenolone into P4 [32]. The results revealed that PLCs showed strong expression of STAR, while the expression of STAR protein was completely absent in P15 and P30 luteal cells (Fig 6) S1 Fig. and S2 Fig.)). Together, these results indicate that with repeated *in vitro* passaging the synthesis of P4 by luteal cells is severely compromised.

### Vimentin and KRT-18 were co-expressed in early to high passaged luteal cells

**Fig 7:**
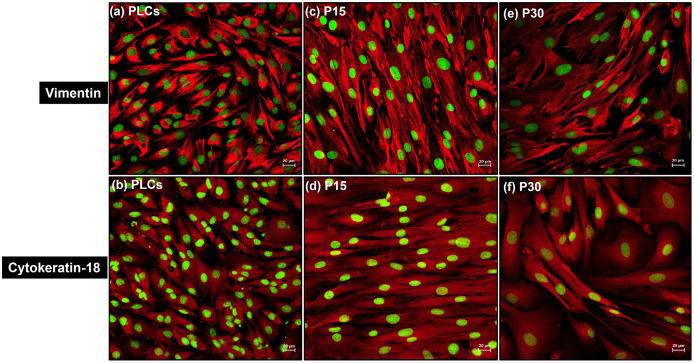
Cell immunofluorescence showed strong positive expression of both vimentin and KRT-18 proteins (red signal) in cell cytoskeleton of (a, b) PLCs, (c, d) P15 and (e, f) P30 luteal cells respectively. Cell nuclei were stained by SyberGreen. Fluorescent images were captured at 20x magnification under LSM fluorescent microscope, scale bar 20µm.

Intracellular expression of cell cytoskeleton marker proteins vimentin and KRT-18 was localized in cultured PLCs, P15 and P30 luteal cells by immunofluorescence. Cultured PLCs, (Fig 7a, 7b), P15 (Fig 7c, 7d) and P30 (Fig 7e, 7f) explicitly expressed both vimentin and KRT18 in their cytoskeleton. In negative controls (without primary antibody, vimentin/KRT18) for all groups only luteal cell nuclei were visualized as stained by SyberGreen (S3 Fig.). The expression of both vimentin and KRT-18 proteins in luteal cell cytoskeleton indicated that the structural integrity of the luteal cells is unaffected by repeated passaging *in vitro*.

### Luteal cells viability is unaltered by passage number

**Fig 8:**
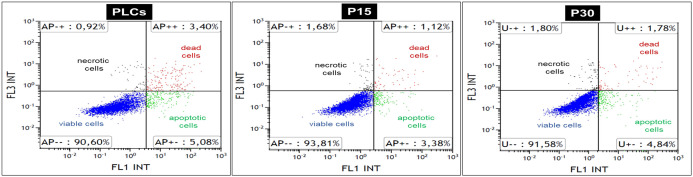
Representative dot plots of Annexin V and propidium iodide (PI) staining results of a single independent experiment in (a) PLCs, (b) P15 and (c) P30 luteal cells generated during single cell flow cytometry analysis. Percentage (%) of cells stained in each quadrant (viable, apoptotic, dead and necrotic luteal cells) are listed in the corner of each quadrant.

Cell viability was analyzed by Annexin/PI staining, followed by flow cytometry to determine the proportions (%) of viable, apoptotic, necrotic and dead luteal cells respectively. Representative quadrat plots of PLCs, P15 and P30 luteal cells are indicated in Figs 8a, 8b and 8c, respectively. Irrespective of passage number, approximately 90% of viability was observed in PLCs, P15 and P30 luteal cells. Similarly, the proportion of apoptotic cells was similar among PLCs, P15 and P30 luteal cells, but the proportion of dead cells was significantly higher (*p < 0.05*) in PLCs compared to P15 and P30 luteal cells. The proportion of necrotic cells were found to be significantly higher (*p < 0.05*) in P30 luteal cells compared to PLCs and P15 luteal cells (Table 2). Together, these results indicate that despite of repeated passaging, a significant proportion of luteal cells remain viable *in vitro* which might be due to activation of several cell survival pathways even at higher passage number.

**Table 2:** Cell viability of *in vitro* cultured PLCs, P15 and P30 luteal cells.

	Viable cells (%)	Apoptotic cells (%)	Dead cells (%)	Necrotic cells (%)
PLCs	90.07 ± 0.35	4.82 ± 0.12	3.96 ± 0.33^a^	1.13 ± 0.15^a^
Passage 15	90.82 ± 1.68	5.71 ± 1.23	1.75 ± 0.45^b^	1.69 ± 0.15^a^
Passage 30	91.03 ± 0.07	4.38 ± 0.40	1.50 ± 0.15^b^	3.11 ± 0.57^b^

Significant changes were recognized with one way ANOVA, if *p < 0.05*. Data represented as means±SEM, n=3.

## Discussion

**Fig 9:**
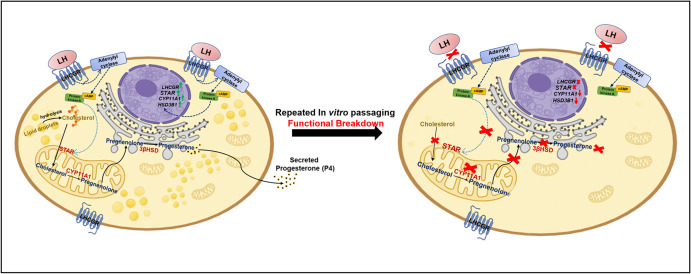
Schematic illustration (created partially with Scientific Image and Illustration Software | BioRender)of possible effects of repeated passaging of cultured luteal cells leading to complete downregulation of functional genes (*STAR*, *HSD3B1* and *LHCGR*) thus completely repressing the crucial function of P4 synthesis of luteal cells. Cholesterol esters stored in lipid droplets are hydrolyzed to free cholesterol. Steroidogenic acute regulatory protein (STAR) aids the transport of free cholesterol from the outer mitochondrial membrane to the inner membrane [53]. Luteal cells respond to luteinizing hormone (LH) activating LHCGR, which further stimulates adenylyl cyclase leading to increases in intracellular cAMP and activation of protein kinase A (PKA). PKA phosphorylates STAR and stimulates cholesterol transport. Once, inside the mitochondria, cholesterol is converted to pregnenolone via P450 side chain cleavage enzyme (CYP11A1). Subsequently pregnenolone is converted into progesterone via 3β-hydroxysteroid dehydrogenase (HSD3B1) present in the endoplasmic reticulum. The secreted P4 then diffuses into the circulation [54,55]. However, with repeated *in vitro* passaging, the lipid content in luteal cells is diminished and the expression of LHCGR is lost leading to complete downregulation of functional genes (*STAR*, *HSD3B1* and *LHCGR*) of luteal cells thus completely repressing the crucial function of P4 synthesis of luteal cells.

The purpose of *in vitro* cell passaging is to maintain the health of cells *in vitro* and simultaneously get a substantial number of cells as ideal *in vitro* model systems for various experimental analyses in basic and medical sciences [33]. Additionally, the sub-cultivation or passaging of a primary culture is the most initial step in establishing a cell line [34], though not all primary culture passaging yields cell lines as most of the cultured cells over a period undergo senescence and eventually die. Another huge limitation of passage is that when primary cells or cell lines are repeatedly passaged for proliferation, they show genotypic and phenotypic variability. Several studies in the past have explored this limitation of using *in vitro* passaged cells. *In-vitro* aging of adult mesenchymal stem cells (MSCs) lead to reduced cell proliferation and morphology at late passage 8 [35,36]. At later passages (P6-P8) the doubling rate increases, which indicates that with passaging cell populations show a decreased proliferation [14,37]. In our study, proliferation of both P15 and P30 luteal cells decreased as compared to PLCs as indicated by the higher proportion of PLCs in S-phase of cell cycle. Contrary to this, higher proportion of P30 cells in G2 phase suggested that at higher passage number luteal cells tend to decrease their proliferative potential and might undergo cell cycle arrest. Cell size enlargement has been associated to cell aging *in vitro* [38]. PLCs displayed a normal elongated cell morphology while with successive passaging both P15 and P30 luteal cells acquired more enlarged and flattened cell morphology. Bovine luteal cells contain abundant lipid droplets which store cholesterol in form of cholesteryl esters for synthesis of P4 [18]. As evident by fluorescent staining, the PLCs were highly loaded with lipid droplets whereas negligible amounts of lipid droplets were observed in P15 and P30 luteal cells. These results were well supported by the negligible P4 concentrations detected in spent media of both P15 and P30 luteal cells compared to very high amounts of P4 observed in PLCs spent media. However, previous studies in bovine endometrial epithelial cells [39], human endometrial stromal cells [40] and porcine oviductal epithelial cells [41] have reported that long-term cultured primary cells at high passage number could still maintain their functional molecular features. Similarly, a report in multipotent murine bone marrow stromal cells D1 cells, revealed that the concentration of triglycerides remains constant even until higher passages (P34) while the expression of osteogenic genes fluctuated significantly among different passages [42]. In the present study, lower mRNA expression of steroidogenic genes *STAR, HSD3B1* and of *LHCGR* in P15 and P30 luteal cells was indicative of a negative correlation of gene expression and passage number. Such alterations in gene expression with passaging have been well documented in various cell types [3,14]. Together, these results suggests that with repeated passaging both lipid droplet formation and expression of key genes regulating the function of P4 synthesis is reduced in luteal cells (Fig 9). In parallel, sequencing data revealed the steroidogenesis and luteal marker genes (*STAR, LHCGR, HSD3B1*) as lowly expressed, but genes regulating ATP generation (*COX1, COX3, ATP8)* and differentiation processes *(SPARC)* [43] as among highly expressed genes in early passaged P3 luteal cells. Additionally, genes such as Y-box binding protein 1(*YXB1*), *VIM*, ubiquitin A-52 residue ribosomal protein fusion product 1(*UBA52*) and several genes encoding ribosomal proteins (RPS) were identified in significant gene clusters that contributed to functions such as “cellular macromolecule biosynthetic process”, “peptide metabolic process”, “translation”, “anatomical structure of morphogenesis” and “tissue development”. Together, these results indicated that with subsequent *in vitro* passaging even at early passage number luteal cells start to show decreased expression of genes associated to steroidogenesis. However, the higher expression of several structural and basic functional genes that are required for cell adhesion, cell proliferation and differentiation during CL formation remains abundantly expressed at least in early passaged P3 luteal cells. Cell cytoskeleton proteins vimentin and KRT-18 are vital for structural integrity of cells [44,45] and interestingly, both vimentin and KRT-18 were found to be consistently expressed in PLCs, P15 and P30 luteal cells. Consistent expression of cell cytoskeleton proteins has also been detected in murine mesothelial cells until high passage 24 [46]. Primary cell cultures and cell lines as *in vitro* cell models for cytotoxicity screening have been widely used [47,48]. However, for cytotoxicity assays, it is critical to know the proportions of live and dead cells present during or after the end of the experiment. This becomes more crucial when cells are cultured for a longer period [49]. An increased susceptibility of human proximal tubular HK-2 cells to toxic compounds such as cadmium chloride (CdCl_2_) was observed at different passages indicating that highly passaged cells could be non-reliable models for screening toxic compounds [50]. Interestingly, in the present study approximately 90% of cultured PLCs, P15 and P30 remained highly viable irrespective of passaging. Although, compared to P15 and P30 luteal cells, higher proportions of dead PLCs were observed which could be because PLCs were isolated directly from CL tissue. Thus, cells were more susceptible to undergo direct cell death due to sudden shift from their strict *in vivo* to *in vitro* environment. On the other hand, necrotic cells appear enlarged due to increased cell volume and thus oversized cells are under stress [51]. Higher proportion of necrotic P30 luteal cells could be indicative of stress imposed on luteal cells due to repeated passaging *in vitro*. Also, excessive cell growth restricts the cells potential to proliferate and induces cellular senescence [35,52]. This could explain the higher proportion of necrotic P30 luteal cells and lower proportion of P30 luteal cells in S-phase of cell cycle. Additionally, a higher proportion (approx. 90%) of PLCs, P15 and P30 luteal cells remained viable despite of repeated passaging *in vitro*, which suggests that proteins and pathways involved in cell survival might remain functional in long term passaged cells. Despite higher passaging, many cell types in *vitro* are able to retain some of their initial characteristics while some specific cell functions like in luteal cells can become highly susceptible to changes *in vitro*. Thus, it is crucial to consider the influence of *in vitro* passaging on the functional characteristics when designing *in vitro* luteal cell-based experiments or new therapeutic strategies.

## Conclusion

Key functional features of specialized cells such as luteal cells are either altered or are completely lost during repeated passaging for propagation *in vitro*. This can potentially affect the experimental outcomes in long-term cultured luteal cells. Therefore, it is highly recommended to define and set the passage number while using luteal and possibly other primary cells in *in vitro* studies.

## Supporting information

S1 File List of gene primers used in RT-qPCR gene expression analysis. (ZIP)

S2 File RNA sequencing gene dataset. (XLSX)

S1 Fig Capillary electrophoresis gel raw image of beta-actin protein. (TIFF)

S2 Fig Capillary electrophoresis gel raw image of STAR protein. (TIFF)

S3 Fig Immunofluorescence image of negative controls (without primary antibody). (TIF)
