# Supplementary material for: Functional characterization of a bovine luteal cell culture model: Effects of passage number
Source: PLoS One. 2025 Nov 19;20(11):e0334047. doi: 10.1371/journal.pone.0334047 (PMC12629482; doi:10.1371/journal.pone.0334047)
Supplement: S1 File — (ZIP) [file pone.0334047.s001.zip › Supplementary sheet 1.docx]

| **Genes** | **Sequence** | **Size (bp)** | **NCBI accession No.** |
| --- | --- | --- | --- |
| ***STAR*** | For. TTGTGAGCGTACGCTGTACCAAG | 236 | NM_174189.2 |
|  | Rev: CTGCGAGAGGACCTGGTTGATG |  |  |
| ***HSD3B1*** | For. TGTTGGTGGAGGAGAAGGATCTG | 208 | NM_174343 |
|  | Rev. GCATTCCTGACGTCAATGACAGAG |  |  |
| ***LHCGR*** | For:GCATCCACAAGCTTCCAGATGTTACGA  Rev: GGGAAATCAGCGTTGTCCCATTGA | 205 | NM_174381 |
|  |  |  |  |
| ***RPLP0*** | For: TGGTTACCCAACCGTCGCATCTGTA  Rev: CACAAAGGCAGATGGATCAGCCAAG | 142 | NM_001012682 |
|  |  |  |  |

List of gene primers used in RT-qPCR gene expression analysis:
