# Supplementary material for: Functional characterization of a bovine luteal cell culture model: Effects of passage number
Source: PLoS One. 2025 Nov 19;20(11):e0334047. doi: 10.1371/journal.pone.0334047 (PMC12629482; doi:10.1371/journal.pone.0334047)
Supplement: S1 File — (ZIP) [file pone.0334047.s001.zip › Supplementary sheet 2.docx]

Calculation of cell doubling time:

| **Passage Number** | **Culture Start** | **Culture End** | **Culture Time (days)** | **Cell doubling time (days)** |
| --- | --- | --- | --- | --- |
| **1** | 300000 | 1215000 | 4 days | 1.982 |
| **3** | 445000 | 975000 | 2 days | 1.767 |
| **4** | 975000 | 5375000 | 7 days | 2.842 |
| **5** | 2687000 | 6420000 | 6 days | 4.775 |
| **6** | 1000000 | 4470000 | 8 days | 3.703 |
| **7** | 1000000 | 2700000 | 11 days | 7.676 |
| **8** | 2700000 | 5400000 | 10 days | 10 |
| **9** | 1500000 | 2450000 | 6 days | 8.477 |
| **11** | 474000 | 1275000 | 10 days | 7.005 |
| **12** | 1275000 | 2640000 | 14 days | 13.33 |
| **13** | 250000 | 795000 | 14 days | 8.388 |
| **14** | 1000000 | 5520000 | 8 days | 3.246 |
| **15** | 330000 | 615000 | 4 days | 4.45 |
| **16** | 25000 | 70000 | 9 days | 6.059 |
| **17** | 30000 | 125000 | 13 days | 6.314 |
| **18** | 62500 | 190000 | 6 days | 3.74 |
| **19** | 70000 | 150000 | 8 days | 7.276 |
| **22** | 130000 | 600000 | 19 days | 8.611 |
| **23** | 600000 | 1350000 | 7 days | 5.983 |
| **24** | 300000 | 1325000 | 17 days | 7.933 |
| **25** | 600000 | 1275000 | 6 days | 5.517 |
| **26** | 637000 | 2050000 | 15 days | 8.895 |
| **27** | 765000 | 1320000 | 12 days | 15.25 |
| **28** | 333000 | 825000 | 12 days | 9.168 |
| **29** | 3800000 | 5850000 | 6 days | 9.64 |
| **30** | 650000 | 960000 | 12 days | 21.33 |

Formula used : Cell doubling time = duration x log(2)

log(Final Concentration)−log(InitalConcentration)

Roth V. 2006 Doubling Time Computing, Available from: http://www.doubling time.com/compute.php
