# Supplementary material for: Functional characterization of a bovine luteal cell culture model: Effects of passage number
Source: PLoS One. 2025 Nov 19;20(11):e0334047. doi: 10.1371/journal.pone.0334047 (PMC12629482; doi:10.1371/journal.pone.0334047)
Supplement: S1 File — (ZIP) [file pone.0334047.s001.zip › Supplementary sheet 3.docx]

RT-qPCR amplicons of LHCGR gene were run on a 3 % agarose gel and the resulting images displaying the quantified LHCGR gene expression in PLCs, P15 and P30 luteal cells respectively.

**Primary luteal cells (PLCs)**


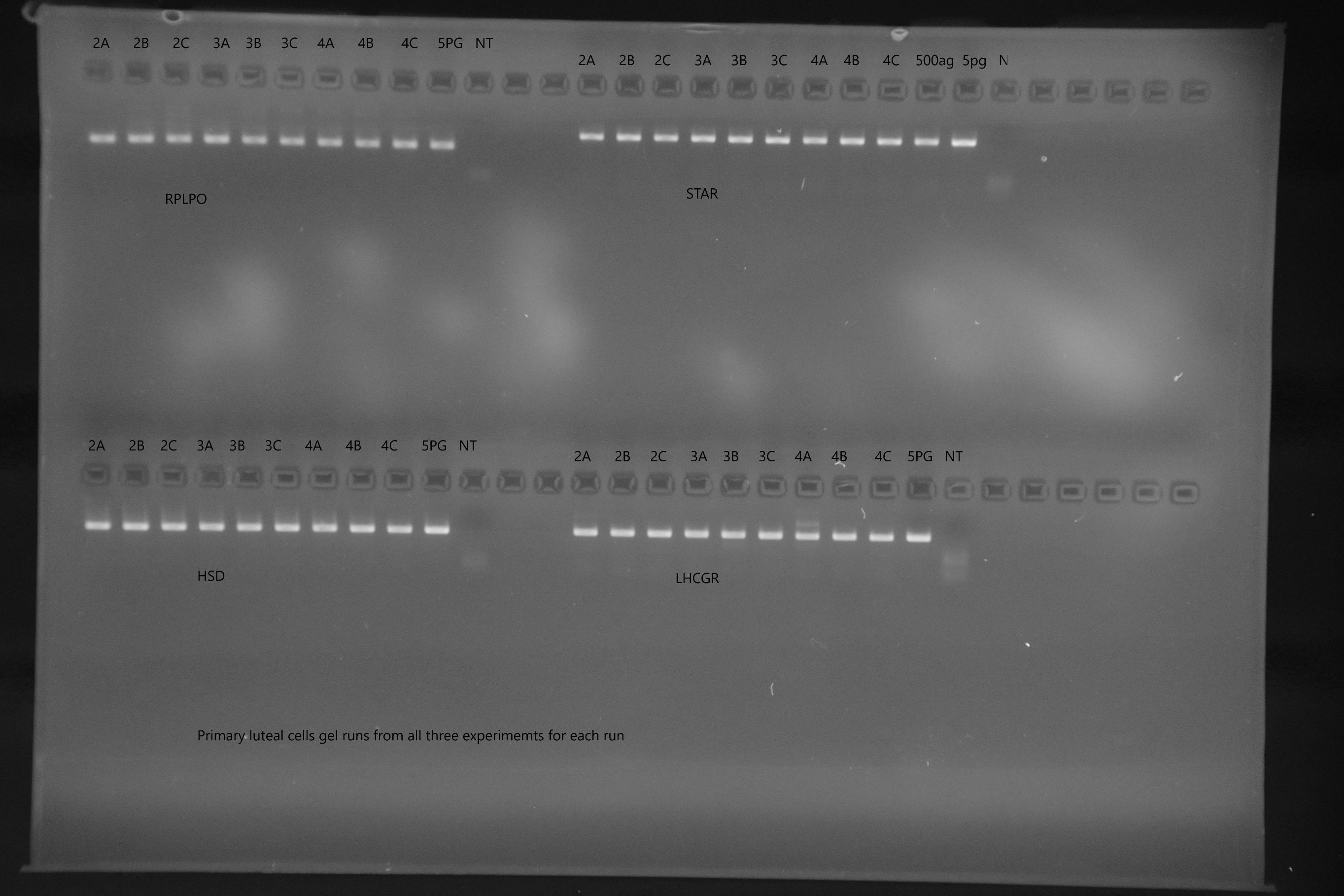


**P15 luteal cells**


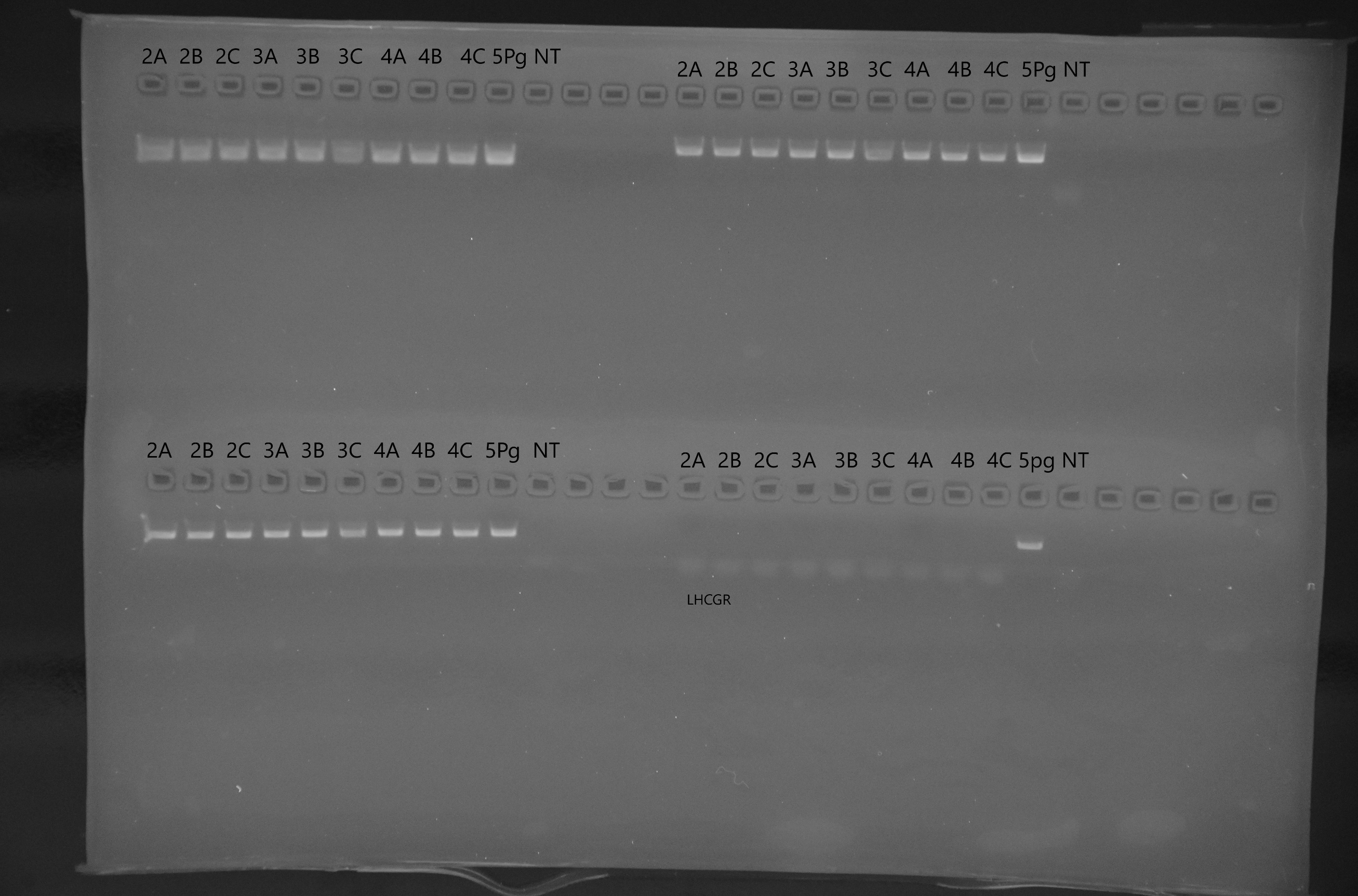


**P30 luteal cells**


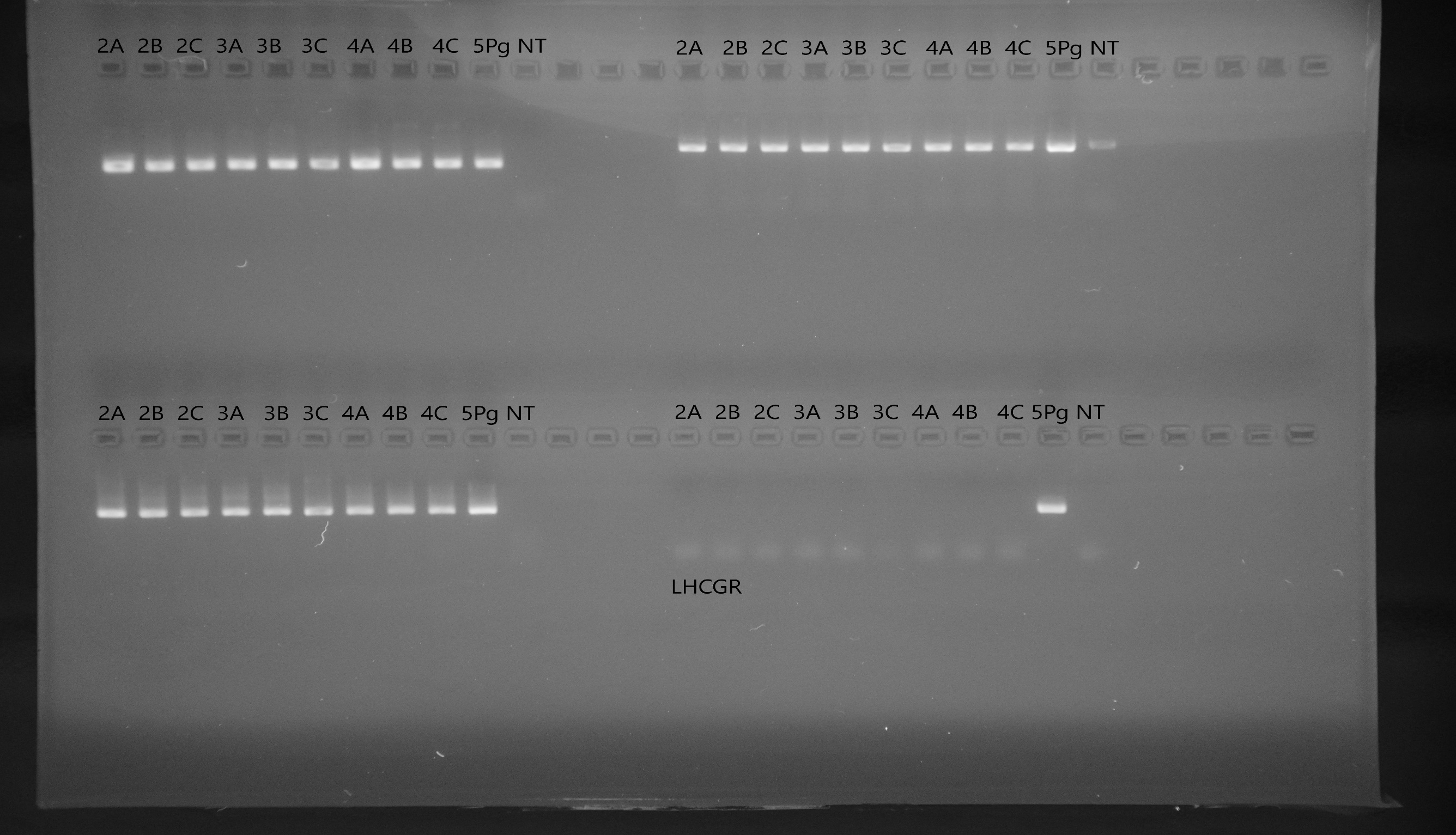


2A, 2B , 2C represent 3 technical replicates from first independent experiment

3A, 3B , 3C represent 3 technical replicates from second independent experiment

4A, 4B , 4C represent 3 technical replicates from third independent experiment

5pg: 5 picogram of internal standard (205 bp)

NT: represent negative control
