# Supplementary figures and images for: Functional characterization of a bovine luteal cell culture model: Effects of passage number
Source: PLoS One. 2025 Nov 19;20(11):e0334047. doi: 10.1371/journal.pone.0334047 (PMC12629482; doi:10.1371/journal.pone.0334047)

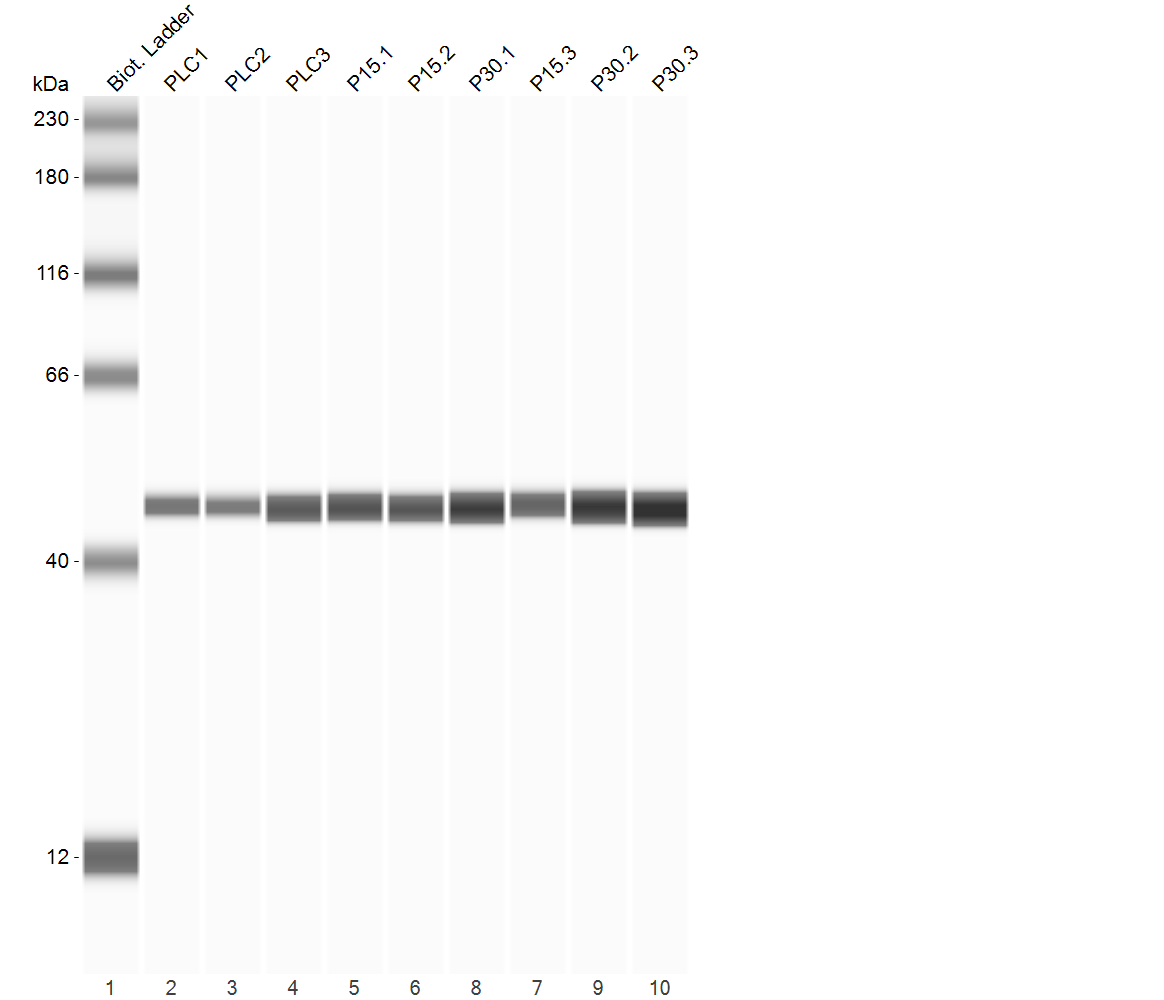

Supplement: S1 Fig — (TIFF) [file pone.0334047.s003.tiff]

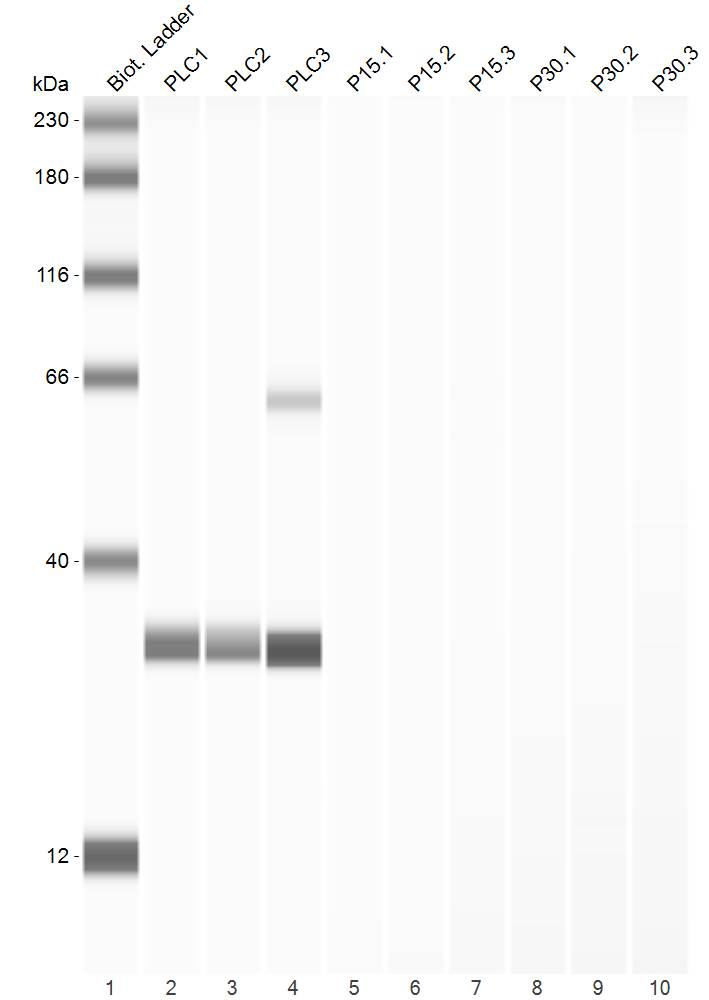

Supplement: S2 Fig — (TIFF) [file pone.0334047.s004.tiff]

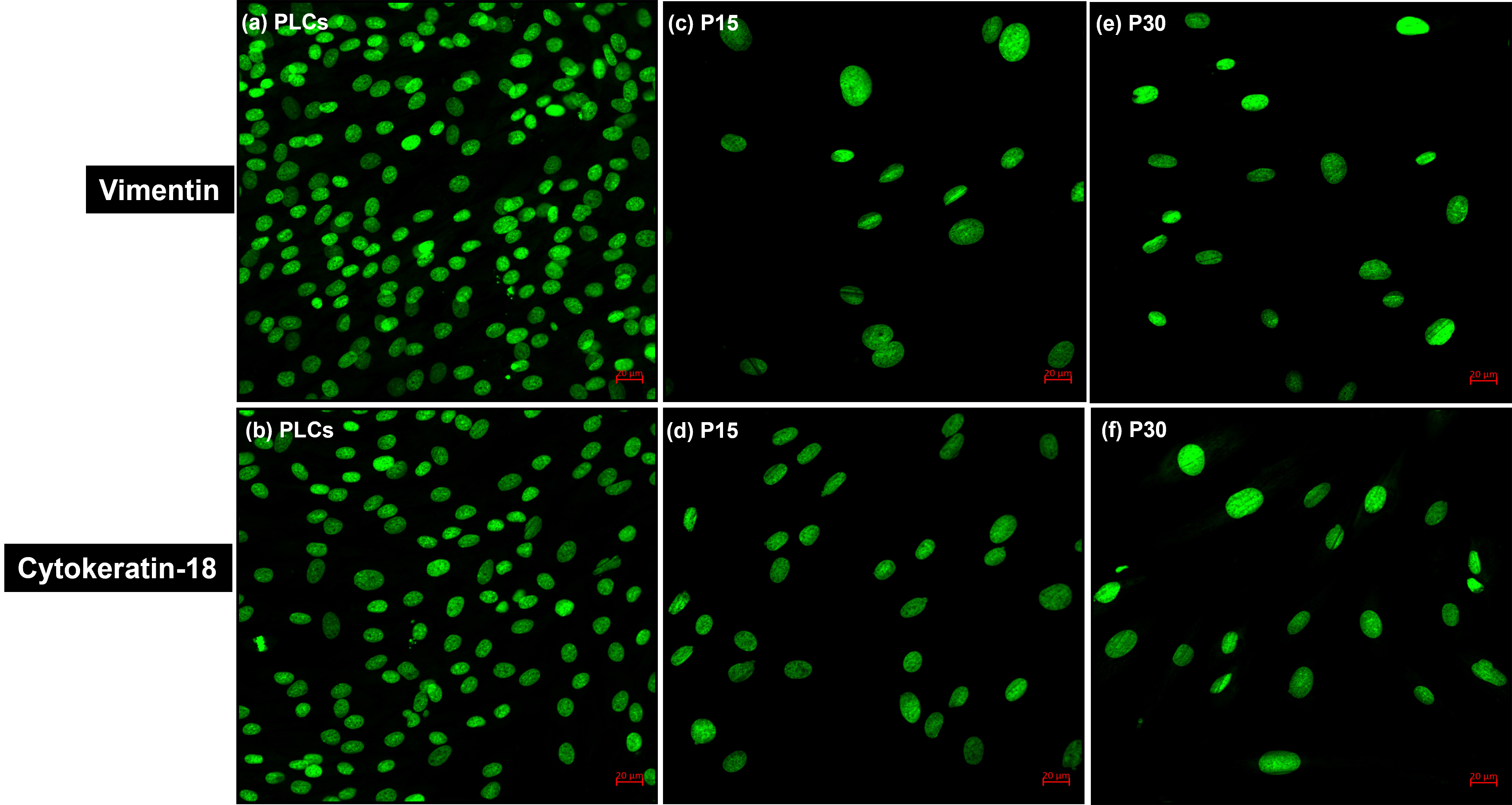

Supplement: S3 Fig — (TIF) [file pone.0334047.s005.tif]
